# Mr. Ring, on the L. Dispensatory—Diarrhæa—Leeches

**Published:** 1804-10-01

**Authors:** John Ring

**Affiliations:** New Street, Hanover Square


### To the Editors of the Medical and Physical Journal.

GENTLEMEN,

IN the last Number of your Journal are some Remarks on the Cow-pock by Dr. Walker; who tells us, that when the variolous pock has acquired its full dimensions, it breaks into a number of distinct vesicles, while the cow-pock, or rather the congeries of vesicles of which it consists, remains united.

This is an inaccurate description of the variolous pustule, excited by inoculation. It does not break into a number of pustules. On the contrary, there is an eruption of secondary pustules near to the primary pustule, with which they become confluent; so that, instead of dividing into many parts, variolous pustules have a tendency to unite.

It is well known, that matter is most active when taken from a vaccine vesicle at an early period; it is also well known, that it will sometimes succeed when diluted, and even when mixed with blood: but I am sorry to find the necessary precautions in taking matter are so much neglected, that one practitioner has seen such matter used a thousand times.

Dr. Walker tells us, that pus taken from under a scab, and used in inoculation, will not produce any effect. Others tell us another tale; and if such practices are encouraged, we shall often hear of the same disastrous consequences as have already happened from the abuse of vaccination.

Dr. Walker tells us, that if the scab be removed, the pus wiped away, and the part underneath broken down with a lancet, it yields, in a smaller quantity, a fluid equally active with that which is taken from an uninterrupted pustule. If such hazardous experiments are ever made, the less that is said about them the better.

Dr. Walker asks, why we should say, *vaccine*, in imitation of the French? The fact is, the French say *vaccine* in imitation of the English. He says, we lay aside the term Cow-pock. This is a mistake; there is no term in more common use.

He considers *vacciola*, the word proposed by Dr. Stokes, the most happy word to be adopted as *a root*; from which all the terms used in the practice may be derived with more advantage. This *root* is *radically* improper. If admitted at all as a Latin word, it is a diminutive from vacca, ca, and of the same import as vaccula, a little cow. Vaccine means something of or belonging to a cow; and is perfectly expressive of the object under consideration. Hence we say, vaccine matter, vaccine virus, the vaccine fluid; vaccine inoculation, the vaccine disease, vaccination; and to vaccinate.

Dr. Walker says, he is sorry to see me follow the French nomenclature in my Treatise. I have the pleasure to inform him, that I have not followed it. The French nomenclature consists of four words; two of them I used before the French used them; the other two I have never used at all.

It is true, I copied the French nomenclature; but at the same time declared it to be, "for the sake of those into whose hands any Treatise on this important branch of medical science, written in the French language, may happen to fall." Dr. Walker has also copied it; but this is by no means an approbation or disapprobation of the terms therein contained.

I thank your Correspondent, S. M. for his information concerning the London Dispensatory; and I think it a duty, on this occasion, to express a wish, that whenever any alterations are made in this work, public notice were given of the circumstance, in order to promote that uniformity in the composition of the medicines therein directed, which is so much to be desired.

Your Correspondent denies the merit of novelty to my observations on diarrhœa, but allows them the much greater merit of being practical and useful. Were every thing but what is novel excluded from your Journal, or any other medical publication whatever, the remainder would lie in a very small compass.

Your Correspondent wishes to know the cause of the great mortality which has lately prevailed among leeches. This, I apprehend, is not to be ascribed to any peculiarity in Thames water, but to the great heat of the summer. Leeches are animals to which the extremes of heat and cold are equally destructive. I have frequently known a similar mortality among them, when they were kept in the New River water.

The wholesale dealers in leeches keep them in spring water during the severity of the winter, because it is then warmer than river water. Possibly, it may be better than river water during the summer, because it is then colder; and particularly if the putrefaction in river water have any any share in producing the mortality in question. It is obvious, that a cool situation should be preferred in summer, and a warm one in winter.

The observations in your last Number, relative to eruptions after vaccination, by Dr. Adams, are interesting; as every thing must be that comes from his pen. Having seen him, when he was in London, I told him that it was the general opinion of those whom I had heard speak on the subject, that the eruptive cases he met with at Madeira were not cases of the cow-pock. I asked him whether he was still of opinion that they were cases of this kind, and he answered in the affirmative.

By a reference to those cases, which were published in the Medical and Physical Journal, for April, 1803, it appears, in some of them the eruption took place without any local sign of infection on the arm. This is so different from what has been observed in other parts of the world in general, that we must be excused for harbouring a certain degree of scepticism on the subject, till the point is cleared up, by the future experiments and observations of other practitioners, with other cow-pock matter.

This scepticism is rather increased than diminished by Dr. Adams's late communication, in which we are informed, that the same event took place in a patient who had the itch. No local effect was produced; and Dr. Adams, in this as well as the other cases, might well be astonished at the appearance of a great number of vesicles on other parts of the body; but it by no means follows, that they were of the vaccine kind.

While we are sceptical with regard to the nature of the eruptions which have occurred in the practice of Dr. Adams, we cannot be hurt at his being sceptical with regard to the eruptions which have occurred in our practice. He suspects, that some of the eruptions which appear at a remote period after vaccination, which the zealots on one side call the small-pox, and those on the other the chicken-pox, are nothing but vaccine vesicles.

I have seen many such cases, and have never seen one of them bear a resemblance to a vaccine vesicle. I have known matter taken from such eruptions produce the chicken-pox. Dr. Willan has also seen several of these cases, and pronounced them to be the chicken-pox; and Dr. Adams will not call Dr. Willan a zealot.

I know no reason to suppose, that eruptions which appear at *a remote period* are owing to vaccine inoculation; nor nor do I know of any new species of vesicular eruption which has lately appeared. Instead, therefore, of our supposed cases of chicken-pox being the cow-pock, I am inclined to think, Dr. Adams's supposed cases of cow-pox were the chicken-pox.

Dr. Adams wishes we were all more attentive to mark phænomena, than hastily to get through difficulties. In this sentiment every friend of science must coincide. But when he tells us, that we ought as much as possible to encourage the objections of the captious, it may be necessary to observe, that after the rigorous ordeal which vaccination has now undergone, such objections can only serve to keep the mind of the public in a continual state of alarm.

He allows, that the objections hitherto started are too light to be taken into account, when compared with the contrary evidence. This being the case, nothing should be encouraged that tends to retard the progress of vaccination, and to prevent the present age from enjoying the full advantage of the practice.

Too much encouragement is given to such objections; too much encouragement is given to unauthenticated reports; and too many periodical publications are ready to receive and circulate any lie that is fabricated against vaccination. One of these lately refused admission to a letter from Dr. Marshall, complaining of the iliberallity of an anonymous attack on the practice and himself; and pledging himself to refute the charges, if the author of the attack would publish his name, and specify the names of the parties in whom the pretended failures had occurred. Admission was also refused to a respectful representation of the impropriety of admitting anonymous attacks of this kind, and anonymous reports on so important a subject.

But admission was readily granted to a report of an unfavourable kind; a report more congenial with the part espoused by the editors of that publication. This report is concerning a child, who lately died of the small-pox after being supposed to have had the cow-pock. Mr. Faithhorn of Kensington, it is there said, can attest the fact. I have enquired of Mr. Faithhorn, and the following certificate will prove, that as far as his testimony is concerned, the report is destitute of foundation.

"It having been stated, that a child of Mr. Meredith, of Kensington, who lately died of the small-pox, had been inoculated for the cow-pock, and regularly gone through the the disease, and that I could testify the truth of this assertion; I hereby declare, that I never saw the child when under vaccination; and, that, from the account given to me by Mrs. Meredith, I am perfectly convinced he never had the cow-pock.

"Mr. Wilson, surgeon, of Shadwell, and Mr. Cockle, surgeon, of High Holborn, saw the child with me; and after making every possible enquiry of the parents, were also of opinion that the child never had the cow-pock.

*Kensington, Sept.* 21, 1804. JOHN FAITHHORN."

In the account published, it is stated that there was a black scab after the pock. This is contrary to the account given by the mother to Mr. Faithhorn, Mr. Cockle, and Mr. Wilson, who first enquired into the subject. She afterwards gave a different account.

It appears, however, even by her confession, that the child was inoculated on Monday, and that Mr. Collurne never saw him but once after, while supposed to be under vaccination, which was on the Thursday following.

The report states, that Mr. Howard of Pimlico, had a share in inoculating the child. This is likewise false; that gentleman never saw the child when under inoculation; and was not in the neighbourhood of London at the time.

Mr. Merriman, another of the pretended witnesses, knows nothing of the matter. The name of Mr. Wilson of Windmill Street, is probably put down by mistake, instead of Mr. Wilson of Shadwell.

Finding the report so incorrect, I have not thought it worth while to call on all the gentlemen whose names are mentioned. I have, however made enquiries of Mr. Collurne, and the mother of the child, and have received the following information:

The child was inoculated by Mr. Collurne, who saw him again three days after. If at that time he formed any decisive opinion respecting the success of inoculation, it certainly was premature; but this will by no means justify an assertion, that the child had the cow-pock.

According to the mother's account, a pustule rose on each arm, but was rubbed of within a few days. It is, therefore, the more likely to have been of the spurious kind; which consists of only one cell, and is more elevated than the genuine cow-pock; and, on both these accounts, is more frequently ruptured. I am, &c.

*New Street, Hanover Square.* JOHN RING.

*Botanical*

